# Transcriptomic analysis reveals that BVDV alters immune and metabolic responses to *Brucella abortus* A19 in RAW264.7 macrophages

**DOI:** 10.3389/fvets.2026.1788225

**Published:** 2026-04-10

**Authors:** Yingjin Chai, Yu Zhang, Jing Zhang, Shengnan Song, Wendong Fu, Xia Zhou, Yidan Zhang, Tingting Lv, Pan Chen, Jiahui Zhang, Hui Zhang, Jia Guo, Zhen Wang

**Affiliations:** 1 College of Animal Science and Technology, Shihezi University, Shihezi, Xinjiang, China; 2 Changji Animal Disease Prevention and Control Center, Changji, China

**Keywords:** *Brucella abortus* A19, BVDV, co-occurrence, immune response, RNA-seq

## Abstract

**Background:**

The co-occurrence of *bovine viral diarrhea virus* (BVDV) infection and brucellosis in cattle is common. However, the effect of BVDV-induced immunosuppression on the efficacy of the *Brucella abortus* (*B. abortus*) A19 vaccine remains unclear. We hypothesized that BVDV infection suppresses the host immune response to the *B. abortus* A19 vaccine by dysregulating key immune-related genes, thereby potentially affecting vaccine immunoprotection. This study aims to explore the possible mechanism by which BVDV infection may influence the cellular response to the *B. abortus* A19 vaccine, as revealed by transcriptome sequencing results.

**Methods:**

To preliminarily assess the impact on immune organs, a mouse model of co-infection with BVDV and *B. abortus* A19 was established. Pathological sections of the spleen of the mice were prepared to examine the impact of BVDV infection on the immune organs of co-infected mice. Subsequently, to investigate the underlying molecular mechanisms, the RAW264.7 mouse macrophage *in vitro* model of co-infection with BVDV and *B. abortus* A19 was constructed, and the comprehensive effect of BVDV on *B. abortus* A19 in RAW264.7 cells was studied using transcriptome analysis.

**Results:**

Compared to the control group, *B. abortus* A19 single infection resulted in 1,926 differentially expressed genes (DEGs), which were enriched in various immune-related signaling pathways. Compared to the *B. abortus* A19 single infection, the mixed infection group of BVDV and *B. abortus* A19 identified 4,810 DEGs, which were widely involved in immune, inflammatory, and metabolism-related pathways. There were 1,047 overlapping DEGs between the two DEG groups, of which 922 DEGs had a reverse expression trend and were involved in immune inflammation and cellular metabolism.

**Conclusion:**

This study reveals that BVDV infection can significantly alter the expression of genes related to immune response, inflammatory response, and cell metabolism in RAW264.7 macrophages. These findings suggest that BVDV may interfere with the cellular response to the *B. abortus* A19 vaccine *in vitro*, clarifying potential molecular mechanisms whereby BVDV might affect vaccine-induced immune protection. This provides a theoretical basis for further validation in bovine models and for optimizing vaccination strategies in at-risk cattle populations.

## 1 Introduction

The incidence and prevalence of epidemic diseases within the cattle industry result in cattle mortality, diminished production performance, and reproductive challenges. This poses serious public health security risks and causes substantial economic losses (1, 2). Zoonotic diseases caused by bacteria, such as Brucella and *Mycobacterium tuberculosis*, cause reproductive issues, including abortion and infertility, in cattle. Furthermore, chronic exposure to these pathogens poses a serious threat to the health and safety of practitioners (3, 4). Viral diseases such as bovine viral diarrhea and infectious bovine rhinotracheitis are the primary causes of bovine respiratory disease syndrome and reproductive disorders, particularly in intensive farming (5, 6). Therefore, elucidating the molecular mechanisms underlying these key pathogens is important for developing effective prevention and treatment measures.

Brucellosis in cattle is a serious zoonotic disease caused by *Brucella abortus*. The main causes of harm to cattle are miscarriages, stillbirths, and reproductive disorders in pregnant cows, which can be transmitted vertically and horizontally to form persistent infections in the population (7, 8). Infected animals and their products are one of the main causes of human brucellosis (9). The *B. abortus* A19 attenuated live vaccine is widely used for the prevention and management of brucellosis in dairy cattle and other livestock, owing to its robust immunological protection and cost-effectiveness (10).

Bovine viral diarrhea virus (BVDV) belongs to the genus *Pestivirus* of the family *Flaviviridae*. Infections can lead to diarrhea, high fever, persistent infection, reproductive failure, mucosal disease, thrombocytopenia, leukopenia, and bleeding syndromes (11–13). Research has demonstrated that co-infection with porcine epidemic diarrhea virus and BVDV can synergistically activate the NF-κB signaling pathway, leading to an increase in inflammatory factors and more severe tissue inflammation (14). In addition, BVDV, together with parainfluenza virus type 3 (PI3), bovine herpesvirus type 1 (Bhv-1), and *Mannheimia haemolytica*, is involved in bovine respiratory disease syndrome and significantly increases morbidity and mortality (15, 16). Jiao et al. (17) identified that the administration of a BVDV-contaminated classical swine fever vaccine resulted in the failure of classical swine fever immunity, suggesting that BVDV poses a potential risk of cross-vaccine interference. Notably, co-infection with Brucella and BVDV has been detected clinically; however, it is currently unclear whether their coexistence affects the immune efficacy of the *B. abortus* A19 vaccine. Considering the central role of the *B. abortus* A19 vaccine in the prevention and control of brucellosis, clarifying the potential interference of BVDV on its immune effect is crucial for ensuring vaccine efficacy and optimizing epidemic prevention and control strategies.

To investigate the effect of BVDV infection on the immune mechanism of the *B. abortus* A19 vaccine, we used RAW264.7 cells as the infection model. RAW264.7 is a macrophage-like cell line. These cells retain multiple key immune functions of primary macrophages, including the expression of pattern recognition receptors (e.g., Toll-like receptors), activation of pathogen-associated molecular patterns, secretion of important inflammatory cytokines, and phagocytosis (18). As a well-characterized and standardized *in vitro* system, the RAW264.7 cell line is widely employed for initial mechanistic studies of host-pathogen interactions, as it allows for high-throughput transcriptional analysis under controlled conditions (19). Therefore, RAW264.7 cells are an optimal cell line for assessing the effect of BVDV on the immune mechanisms of the *B. abortus* A19 vaccine. This study established RAW264.7 cell models that underwent single infection with *B. abortus* A19 and co-infection with BVDV and *B. abortus* A19. Transcriptome sequencing was performed to assess the effect of BVDV infection on cellular gene expression. This study contributes to the understanding of the potential molecular interference of BVDV infection on the cellular response to the *B. abortus* A19 vaccine, providing a mechanistic basis for future *in vivo* validation.

## 2 Materials and methods

### 2.1 *Brucella abortus* A19 vaccine strain, BVDV strain, and animals

The *B. abortus* A19 vaccine strain was from the Xinjiang Center for Disease Control and Prevention. The CP-type BVDV virus used in this study, BVDV-1b subgenotype, was provided by Fu Qiang of the School of Veterinary Medicine, Xinjiang Agricultural University. The BVDV strain was propagated according to a standard protocol in Madin Darby bovine kidney cells that had been tested and were free of BVDV and HoBi-like viruses (20). Five-week-old female SPF C57BL/6J mice were obtained from Henan Skebes Biotechnology Co., Ltd., and housed at the Animal Hospital of Shihezi University.

### 2.2 Animal handling

The CP BVDV-1b subgenotype used in this experiment and the sample size (*n* = 6) were selected based on previously established methods (21). A total of 24 mice were used in this study. All the animals were healthy and in good physical condition. After 1 week of environment acclimation, the mice were randomly assigned to the following treatment groups: unvaccinated control group (control group, *n* = 6), BVDV single-infection group (BVDV group, *n* = 6), *B. abortus* A19 single-infection group (*B. abortus* A19 group, *n* = 6), and BVDV and *B. abortus* A19 co-infection group (BVDV+A19, *n* = 6). At the time of grouping, it was ensured that the average initial body weight showed no statistically significant differences across the treatment groups. The experimental mice were housed in independent ventilated cages. Strict control of environmental parameters: temperature 22 ± 1 °C, humidity 55 ± 10%, 12/12 h light dark cycle. The cage is equipped with sterile corn cob padding (changed twice a week). All mice are free to obtain sterilized pellet feed and acidified drinking water. All operations follow the “3R” principle and have been reviewed and approved by the Institutional Animal Ethics Committee (protocol code A2025-1073).

The *B. abortus* A19 vaccine strain (200 μL, colony forming unit [CFU] = 10^5^ per mouse) was administered according to the manufacturer’s recommendations, with a BVDV dose of 600 μL (Tissue Culture Infective Dose 50% [TCID50], 10^6.76^ TCID₅₀/mL) via intraperitoneal injection. At the 4th and 8th week post-infection with the *B. abortus* A19 vaccine strain, mice were deeply anesthetized by placing them in a transparent induction chamber with a continuous flow of 5% isoflurane (v/v) in oxygen (flow rate: 1–2 L/min) until loss of righting reflex and absence of response to a toe pinch. Following anesthesia, each mouse was immediately removed from the chamber and euthanized by rapid cervical dislocation performed by a trained researcher. Death was confirmed by the cessation of respiration and heartbeat, after which spleen tissue samples were randomly collected. After HE staining, all spleen slices were systematically evaluated under light microscopy by two pathologists who were unaware of the experimental grouping.

All animal experiments in this study strictly complied with the “Guidelines for the Welfare and Ethical Review of Experimental Animals” and “Guidelines for the Management and Use of Experimental Animals,” and were conducted in accordance with the regulations established by the Experimental Animal Ethics Committee of Shihezi University. All experimental animals were approved by the Experimental Animal Ethics Committee of Shihezi University.

### 2.3 Preparation and testing of transcriptome sequencing samples

RAW264.7 cells were grown in Dulbecco’s Modified Eagle’s medium (DMEM) supplemented with 10% fetal bovine serum (Gibco, Australia) and 1% penicillin/streptomycin at 37 °C with 5% carbon dioxide. The cells were seeded onto a six-well plate and cultured until 70% confluence, and treatment was performed on different infection groups in logarithmic cycles. For the BVDV single-infection group, RAW264.7 cells were cultured in DMEM medium (MOI = 1:1) (22) without fetal bovine serum and 1% penicillin/streptomycin for 2 h. After washing with phosphate-buffered saline (PBS) three times, the maintenance medium was replaced, and the culture was continued for a further 24 h (DMEM supplemented with 2% fetal bovine serum and 1% penicillin/streptomycin). *B. abortus* A19 single-infection group, RAW264.7 cells were cultured with *B. abortus* A19 (multiplicity of infection [MOI] = 100:1) (23) in a medium without fetal bovine serum and 1% penicillin/streptomycin for 1 h. After washing with PBS three times, the maintenance medium was replaced, and the culture was continued for a further 24 h. For the BVDV+A19 co-infection group, RAW264.7 cells were cultured in BVDV (MOI = 1:1) medium without fetal bovine serum and 1% penicillin/streptomycin for 2 h. After washing three times with PBS, the culture medium was maintained for 24 h. After washing three times with PBS, *B. abortus* A19 (MOI = 100:1) was cultured for 1 h in medium without fetal bovine serum and 1% penicillin/streptomycin. After washing with PBS three times, the maintenance medium was replaced, and the cells were incubated for another 24 h. After washing three times with PBS, 1 mL of Trizol was added to each well of the six-well plate to lyse the cells, and the lysate from each well was transferred to individual RNase-free Eppendorf tubes. According to the requirements of Xinjiang Shadow Biotechnology Co., Ltd., the samples were frozen in liquid nitrogen and sent to a sequencing company on dry ice for transcriptome sequencing.

### 2.4 Library construction and data quality control

Following successful assessment using an Agilent 2100 Bioanalyzer, RNA samples with high integrity (RIN ≥ 7) were used for library construction. mRNA was enriched from total RNA using Oligo(dT) magnetic beads (NEBNext® Poly(A) mRNA Magnetic Isolation Module, New England BioLabs). Strand-specific RNA-seq libraries were then prepared using the NEBNext® Ultra™ II Directional RNA Library Prep Kit for Illumina® (New England BioLabs) according to the manufacturer’s protocol. Briefly, the enriched mRNA was fragmented and used for first-strand cDNA synthesis with random primers and M-MuLV reverse transcriptase. Second-strand synthesis was performed in the presence of dUTP to retain strand information. After end repair, A-tailing, and adapter ligation, the dUTP-marked second strand was degraded by the USER enzyme, and the library was PCR amplified and purified using AMPure XP beads (Beckman Coulter). Library quality was assessed using two parameters: (1) concentration was measured by Qubit fluorometer (≥1 nM) and further validated by quantitative PCR (qPCR) to ensure sufficient effective molar mass; (2) fragment size distribution was examined on an Agilent 2100 Bioanalyzer, with a distinct peak at 150–200 bp and no adapter dimers. Only libraries meeting these criteria proceeded to sequencing.

Subsequently, Illumina sequencing was performed on the library that met the quality criteria. The image data of the sequencing fragments measured by the high-throughput sequencer were converted into sequence data (reads) through CASAVA base recognition, with the file in fastq format, which mainly contains the sequence information of the sequencing fragments and their corresponding sequencing quality information. To ensure the quality and reliability of the data analysis, the reads were filtered to remove connectors, N content, and low-quality raw data. The clean reads were quickly and accurately aligned after quality control with the reference genome using HISAT2 software to obtain the localization information of the reads on the reference genome (24).

### 2.5 Analysis of differentially expressed genes

The fragments per kilobase of transcript per million mapped reads (FPKM) was used to calibrate and quantify the sequencing depth and gene length (25). The DESeq2 R software package was used to analyze differentially expressed genes (DEGs). When genes met the difference criteria (padj ≤ 0.05, |log2FoldChange| ≥ 1.0), they were considered to have significant difference. Based on the Kyoto Encyclopedia of Genes and Genomes (KEGG) database,^1^ KEGG analysis was performed on the DEGs using NovoMagic.^2^ A protein–protein interaction (PPI) network was constructed using the STRING database.^3^ The list of immune-related genes was obtained from the InnateDB^4^ and ImmPort^5^ databases.

### 2.6 Quantitative real-time PCR

The extracted RNA was converted to cDNA using a HiFiScript cDNA Synthesis Kit (CWBIO, Jiangsu, China). Primers were designed using Primer PremierTM 5.0 (Sigma Aldrich, St. Louis, MO, United States; Table 1). A real-time PCR Instrument (Thermo Scientific, Waltham, MA, United States) was used with UltraSYBR Mixture (CWBIO) for quantification. The thermal cycling conditions were as follows: initial denaturation at 95 °C for 10 min, followed by 45 cycles of 95 °C for 10 s, 57 °C for 30 s, and 72 °C for 40 s. The results were analyzed using the 2 − ΔΔCT method, with β-actin (β-non-muscle) as a reference gene (26).

**Table 1:** Primers used for qRT-PCR.

Genes	Primer direction	Sequence (5′ → 3′)	Size (bp)	GenBank Number
*Irf7*	Forward	GAGACTGGCTATTGGGGGAG	102	NM_001252600.1
	Reverse	GACCGAAATGCTTCCAGGG		
*Irf9*	Forward	GCCGAGTGGTGGGTAAGAC	200	NM_008394.3
	Reverse	GCAAAGGCGCTGAACAAAGAG		
*Fosl1*	Forward	ATGTACCGAGACTACGGGGAA	140	NM_010235.2
	Reverse	CTGCTGCTGTCGATGCTTG		
*Fos*	Forward	CGGGTTTCAACGCCGACTA	166	NM_010234.3
	Reverse	TTGGCACTAGAGACGGACAGA		
*Fosb*	Forward	TTTTCCCGGAGACTACGACTC	174	NM_001347586.1
	Reverse	GTGATTGCGGTGACCGTTG		
*Runx3*	Forward	CAGGTTCAACGACCTTCGATT	103	NM_001369050.1
	Reverse	GTGGTAGGTAGCCACTTGGG		
*Nfyc*	Forward	GGCAGCCCAGATTTTTATCACT	165	NM_001048168.3
	Reverse	GGAGGTTTCAGTTCATCTCTTGG		
*Nr1h3*	Forward	CTCAATGCCTGATGTTTCTCCT	150	NM_001177730.1
	Reverse	TCCAACCCTATCCCTAAAGCAA		
*Nfatc1*	Forward	GACCCGGAGTTCGACTTCG	97	NM_001164109.1
	Reverse	TGACACTAGGGGACACATAACTG		
*Gata3*	Forward	CTCGGCCATTCGTACATGGAA	134	NM_001355110.2
	Reverse	GGATACCTCTGCACCGTAGC		
*Plscr1*	Forward	GGTATCCCCCTCCGTATCCAC	155	NM_001410453.1
	Reverse	GCCACCACCTGCATAACCT		
*Jun*	Forward	CCTTCTACGACGATGCCCTC	102	NM_010591.2
	Reverse	GGTTCAAGGTCATGCTCTGTTT		
*β-actin*	Forward	TGCTATGTTGCTCTAGACTTCG	240	NM_007393.5
	Reverse	GTTGGCATAGAGGTCTTTACGG		

### 2.7 Statistical analysis

All statistical analyses were conducted using GraphPad Prism software (version 9.0.0). Descriptive data are presented as mean ± SD. For group comparisons, effect estimates are reported with 95% confidence intervals (95% CIs), and a *p* value < 0.05 was considered statistically significant. This study minimizes environmental and time-dependent confounding by regularly rotating cage positions and balancing the intergroup order of daily processing. Establish inclusion criteria (complete intervention and complete data collection) and exclusion criteria (death or sample contamination during the experiment) in advance according to the experiment. During the experiment, all mice in each treatment group survived. The final sample sizes for each group are: control group *n* = 6, BVDV group *n* = 6, *B. abortus* A19 single-infection group *n* = 6, BVDV+A19 co-infection group *n* = 6. When analyzing pathological sections of the spleen, analysts are in a blind state without knowing the grouping information.

## 3 Results

### 3.1 Co-infection of BVDV and *Brucella abortus* A19 vaccine strain exacerbates pathological spleen damage in mice

During the experiment, the clinical symptoms of the infected group of mice were monitored daily. Compared with the control group, mice in the BVDV single-infection group, the *B. abortus* A19 single-infection group, and the BVDV+A19 co-infection group showed clumping and rough fur from the third day after infection. No death was observed throughout the entire experiment.

**Figure 1:**
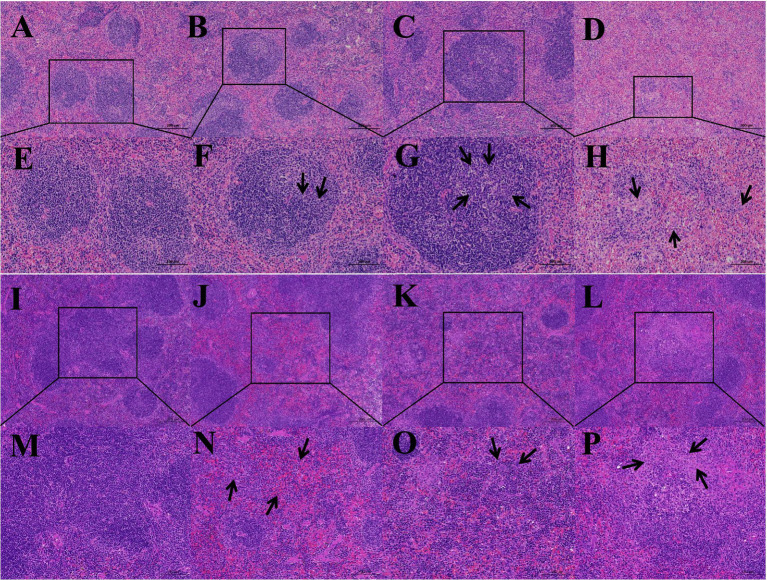
HE staining image of mouse spleen pathological section. **(A–D)** Are the pathological sections of the spleen at 100x magnification on day 28 for the control group, BVDV single infection group, *B. abortus* A19 single infection group, and BVDV+A19 co-infection group, respectively, **(E–H)** are the images of the corresponding groups at 200x magnification on the 28th day, **(I–L)** are the corresponding group images at 100× magnification on day 56, **(M–P)** are pathological sections of the spleen at 200× magnification on day 56. Arrows indicate the location of lesions.

To evaluate the pathogenicity of BVDV+A19 co-infection in mice, pathological sections were prepared from the spleens of mice on days 28 and 56 following infection with the *B. abortus* A19 vaccine strain. Hematoxylin and eosin staining results showed that on day 28, the spleen structure of the BVDV single-infection group and the *B. abortus* A19 single-infection group (Figures 1B,C,F,G) differed from that of the control group (Figures 1A,E), and varying degrees of lymphocyte proliferation were observed in the white pulp area. The BVDV+A19 co-infection group (Figures 1D,H) showed a more obvious degree of proliferation, and the red pulp area was congested. On day 56, Compared with the control group (Figures 1I,M), the BVDV single-infection group and the *B. abortus* A19 single-infection group exhibited pathological changes, including the disappearance of the red and white pulp boundaries and lymphocyte proliferation (Figures 1J,K,N,O). Histopathological analysis of the spleen in the BVDV+A19 co-infection group (Figures 1L,P) revealed severe damage, with a total semi-quantitative score of 8/12. Prominent features included structural disruption (score 3), increased cellular atypia (score 3), and focal hemorrhage with moderate inflammation (score 2), consistent with extensive lymphocytic necrosis. The experimental results showed that BVDV+A19 co-infection caused severe damage to mice spleen, with lymphocyte necrosis as the main lesion.

### 3.2 Transcriptome sequencing obtains high-quality data and achieves efficient genome alignment

A large amount of high-quality raw data was obtained using the Illumina high-throughput sequencing platform to sequence the cDNA libraries. After quality control filtering to remove adapter sequences, N-supplemented, and low-quality reads, clean reads were used for transcript assembly. The amount of clean data in the samples ranged from 41,584,728 to 57,060,884, with Q30 being the lowest at 95.86% (Table 2). The proportion of reads that successfully aligned with the reference genome ranged from 62.86 to 86.58% (Table 3).

**Table 2:** Sequencing data statistics.

Sample	clean_reads	clean_bases	GC_pct	% ≥ Q30
PBS_1	45,092,996	6.76G	50.52	96.7
PBS_2	46,378,778	6.96G	50.32	96.82
PBS_3	44,187,464	6.63G	50.18	96.64
BVDV_1	47,897,924	7.18G	50.2	96.76
BVDV_2	39,658,710	5.95G	50.58	96.58
BVDV_3	44,578,538	6.69G	45.9	95.86
A19_1	47,298,902	7.09G	50.43	96.82
A19_2	57,060,884	8.56G	50.47	96.65
A19_3	49,361,024	7.4G	50.77	96.79
BVDV_A19_1	41,584,728	6.24G	50.19	96.68
BVDV_A19_2	43,608,572	6.54G	50.42	96.79
BVDV_A19_3	47,042,952	7.06G	50.42	96.76

PBS, BVDV, A19 and BVDV_A19 indicate the RAW264.7 in the control, BVDV, *B. abortus* A19 and BVDV +A19 groups, and 1–3.

**Table 3:** Gene comparison efficiency statistics.

Sample	Total_map	Unique_map	Multi_map
PBS_1	36,638,227 (81.25%)	35,180,185 (78.02%)	1,458,042 (3.23%)
PBS_2	37,160,403 (80.12%)	35,675,482 (76.92%)	1,484,921 (3.2%)
PBS_3	34,746,271 (78.63%)	33,446,909 (75.69%)	1,299,362 (2.94%)
BVDV_1	39,038,763 (81.5%)	37,405,554 (78.09%)	1,633,209 (3.41%)
BVDV_2	32,222,256 (81.25%)	30,868,487 (77.84%)	1,353,769 (3.41%)
BVDV_3	28,021,249 (62.86%)	26,737,521 (59.98%)	1,283,728 (2.88%)
A19_1	38,712,262 (81.85%)	36,899,968 (78.01%)	1,812,294 (3.83%)
A19_2	46,919,353 (82.23%)	44,712,966 (78.36%)	2,206,387 (3.87%)
A19_3	41,033,639 (83.13%)	39,152,337 (79.32%)	1,881,302 (3.81%)
BVDV_A19_1	35,777,010 (86.03%)	34,418,857 (82.77%)	1,358,153 (3.27%)
BVDV_A19_2	37,757,122 (86.58%)	36,258,017 (83.14%)	1,499,105 (3.44%)
BVDV_A19_3	40,620,199 (86.35%)	38,974,703 (82.85%)	1,645,496 (3.5%)

PBS, BVDV, A19 and BVDV_A19 indicate the RAW264.7 in the control, BVDV, *B. abortus* A19 and BVDV +A19 groups, and 1–3 indicate the three replicates, respectively. Total_map: percentage of clean reads mapped to the reference genome (including both unique and multiple mappings), Unique_map: percentage of clean reads mapped to a unique genomic location (used for subsequent gene expression analysis), Multi_map: percentage of clean reads mapped to multiple genomic locations (excluded from downstream analysis).

### 3.3 BVDV co-infection exacerbates the transcriptome response of RAW264.7 cells induced by *Brucella abortus* A19 infection

**Figure 2:**
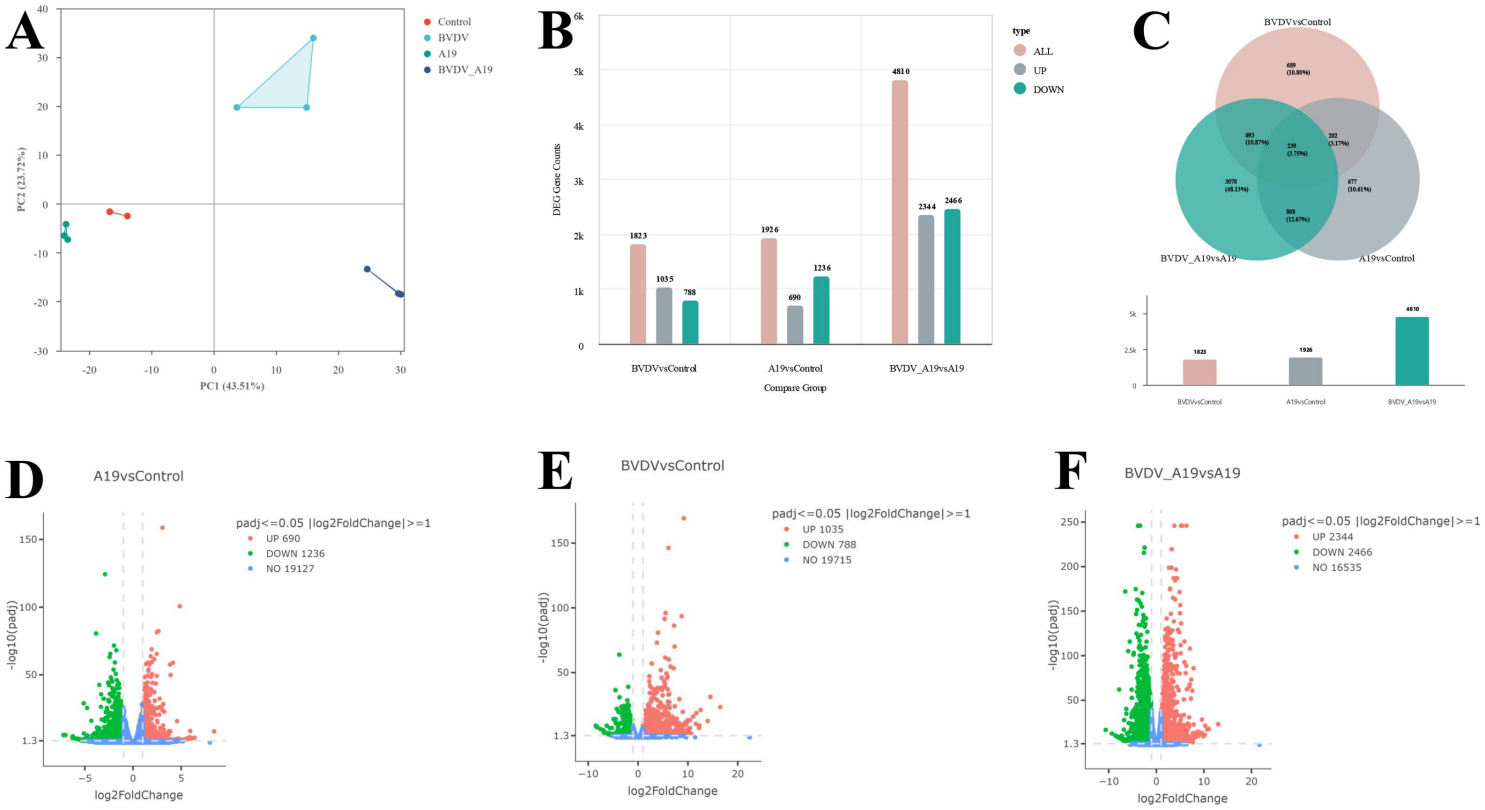
Statistical analysis of transcriptome DEGs. **(A)** Is the PCA plot, **(B)** is the DEGs statistical analysis plot, **(C)** is the Wien analysis plot for three comparison groups of DEGs, and **(D,E,F)** is the volcano plot for DEGs analysis of different comparison groups.

To assess the overall variation between samples and the reproducibility within groups, principal component analysis (PCA) was performed on the normalized gene expression data. The PCA results (Figure 2A) indicate that samples from the same group clustered together, while distinct separation was observed between different treatment groups, indicating good biological reproducibility and clear group-specific expression patterns. The first two principal components explained 43.51 and 23.72% of the total variance, respectively.

To visualize the overall distribution of differentially expressed genes (DEGs) across experimental groups, volcano plots were generated for the most biologically relevant pairwise comparisons (Figures 2D–F). Genes with |log2FC| ≥ 1 and adjusted *p*-value < 0.05 were considered significantly differentially expressed and are highlighted in red (upregulated) and green (downregulated).

Compared with the control group (Figure 2D), the *B. abortus* A19 single-infection groups exhibited 690 upregulated and 1,236 downregulated genes, the BVDV single-infection group (Figure 2E) revealing 1,035 upregulated and 788 downregulated genes. Similarly, the comparison between the BVDV+A19 co-infection group and the A19 single-infection group (Figure 2F) identified 2,344 upregulated and 2,466 downregulated genes, highlighting the transcriptional impact of BVDV co-infection on the host response to A19.

The results of the DEGs analysis (Figure 2B) revealed 1,926 DEGs between the control and *B. abortus* A19 single-infection groups, of which 690 were significantly upregulated, and 1,236 were significantly downregulated. In comparison between the BVDV single-infection group and the control, 1,823 DEGs were identified, of which 1,035 were significantly upregulated and 788 were significantly downregulated (Figure 2B). There were 4,810 DEGs between the *B. abortus* A19 single-infection and BVDV+A19 co-infection groups, of which 2,344 were significantly upregulated, and 2,466 were significantly downregulated (Figure 2B). Venn analysis revealed 239 common genes between the three groups (Figure 2C).

### 3.4 *Brucella abortus* A19 triggers RAW264.7 cell response by activating key immune pathways and regulating key genes

**Figure 3:**
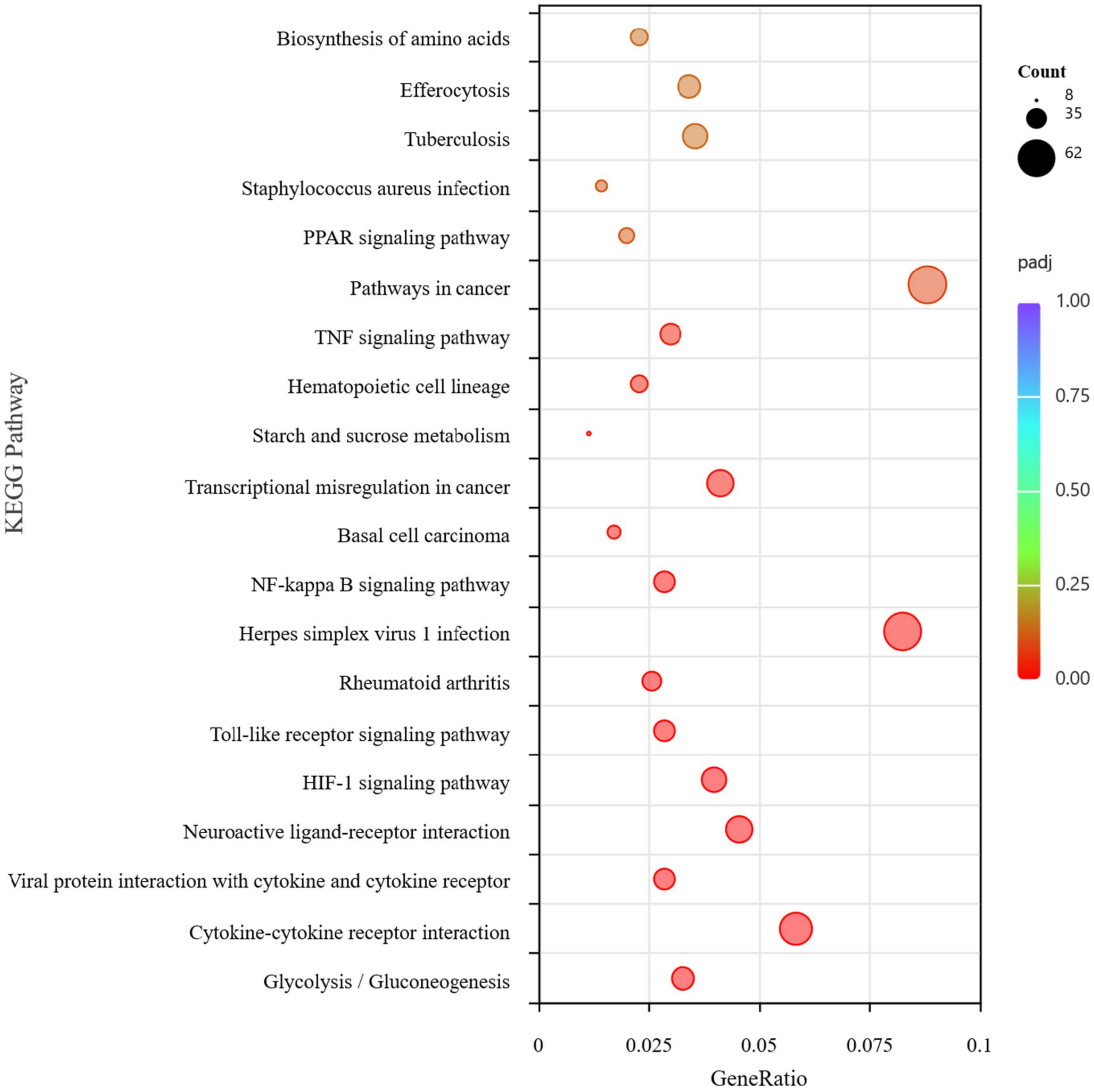
KEGG pathway analysis of DEGs between the control group and the *B. abortus* A19 single infection group.

**Figure 4:**
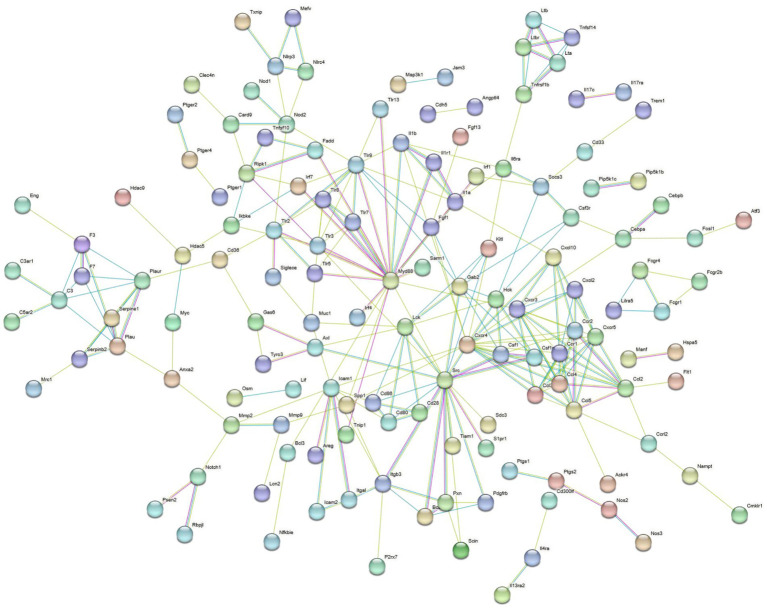
PPI analysis of immune related DEGs in the control group and the *B. abortus* A19 single infection group. The network was generated using the STRING database (https://string-db.org/).

To clarify the function of the DEGs, we conducted KEGG annotation and enrichment analyses of DEGs between the *B. abortus* A19 single-infection and control groups. KEGG signaling pathway analysis showed that the DEGs between the two groups were significantly enriched in the immune pathway, including cytokine-cytokine receptor interaction and Toll-like receptor, NF-kappa B, and TNF signaling pathways. Therefore, the *B. abortus* A19 vaccine strain induced an immune response in RAW264.7 cells to eliminate the invasion of *B. abortus* A19 (Figure 3). A list of genes involved in the immune signaling pathway is presented in Table 4. Genes related to the immune response included *Ccr1* (C-C chemokine receptor type 1), *Tlr7* (Toll-like receptor 7), *Cd86* (Cluster of Differentiation 86), *Il10* (Interleukin-10), *Tbx21* (T-box transcription factor), *Nod2* (nucleotide-binding oligomerization domain-containing protein 2), *Ikbke* (inhibitor of nuclear factor kappa B kinase subunit epsilon), and *Irf7* (interferon regulatory factor 7). Immune-related gene screening was conducted on 1,926 DEGs using the InnateDB, ImmPort, and KEGG Immune System databases, of which, 240 DEGs were identified. PPI network analysis was performed on these 240 immune-related DEGs. The genes *Myd88* (myeloid differentiation primary response protein), *Cxcr4* (C-X-C chemokine receptor type 4), *Icam1* (intercellular adhesion molecule 1), and *Ccl5* (C-C motif chemokine 5) may serve as key genes for the response of RAW264.7 cells to *B. abortus* A19 infection (Figure 4).

**Table 4:** PPI analysis of immune related DEGs in the control group and the *B. abortus* A19 single infection group.

KEGG pathway	KEGGID	Gene name	*p-*value
Cytokine-cytokine receptor interaction	mmu04060	*Ccr1, Csf1r, Il16, Il4ra, Il6ra, Cxcr3, Ltbr, Il10rb, Tnfrsf1b, Ltb, Tnfsf14, Crlf2, Cxcr5, Il1r1, Csf3r, Il17ra, Il1b, Ccr2, Cxcl2, Osm, Cxcr4, Ccl3, Il27, Gm16712, Il21r, Lif, Il23a, Il17c, Ccl2, Csf1, Il13ra2, Mir7676-2, Il1a, Ccl4, Tnfsf10, Ccl5, Cxcl10, Lta*	0.0000003
Toll-like receptor signaling pathway	mmu04620	*Tlr2, Tlr7, Tlr8, Tlr9, Cd86, Fadd, Tlr3, Irf7, Cd80, Myd88, Ripk1, Ikbke, Tlr5, Il1b, Map2k6, Ccl3, Ccl4, Spp1, Ccl5, Cxcl10*	0.0001053
NF-kappa B signaling pathway	mmu04064	*Gadd45b, Ptgs2, Traf1, Icam1, Ltbr, Ltb, Myd88, Ripk1, Tnfsf14, Il1r1, Gadd45a, Il1b, Cxcl2, Gadd45g, Plau, E230016M11Rik, Lck, Ccl4, Lta*	0.0002763
TNF signaling pathway	mmu04668	*Mmp9, Ptgs2, Traf1, Icam1, Fadd, Tnfrsf1b, Bcl3, Cebpb, Ripk1, Nod2, Irf1, Il1b, Map2k6, Cxcl2, Socs3, Lif, Ccl2, Csf1, Ccl5, Cxcl10, Lta*	0.0016837

### 3.5 BVDV triggers RAW264.7 immune response via key immune pathways and core genes

**Figure 5:**
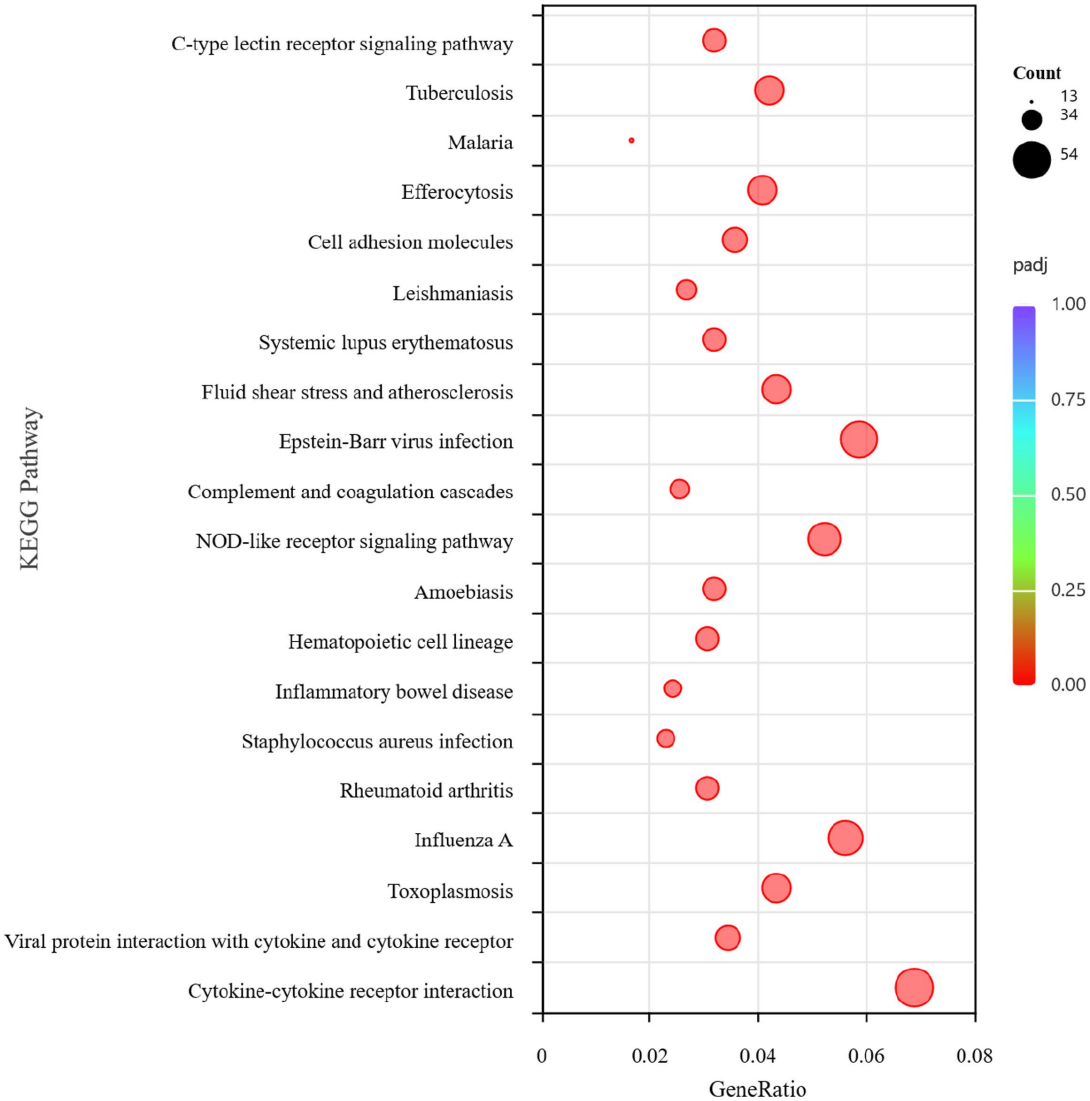
KEGG pathway analysis of DEGs between the control group and the BVDV single infection group.

**Figure 6:**
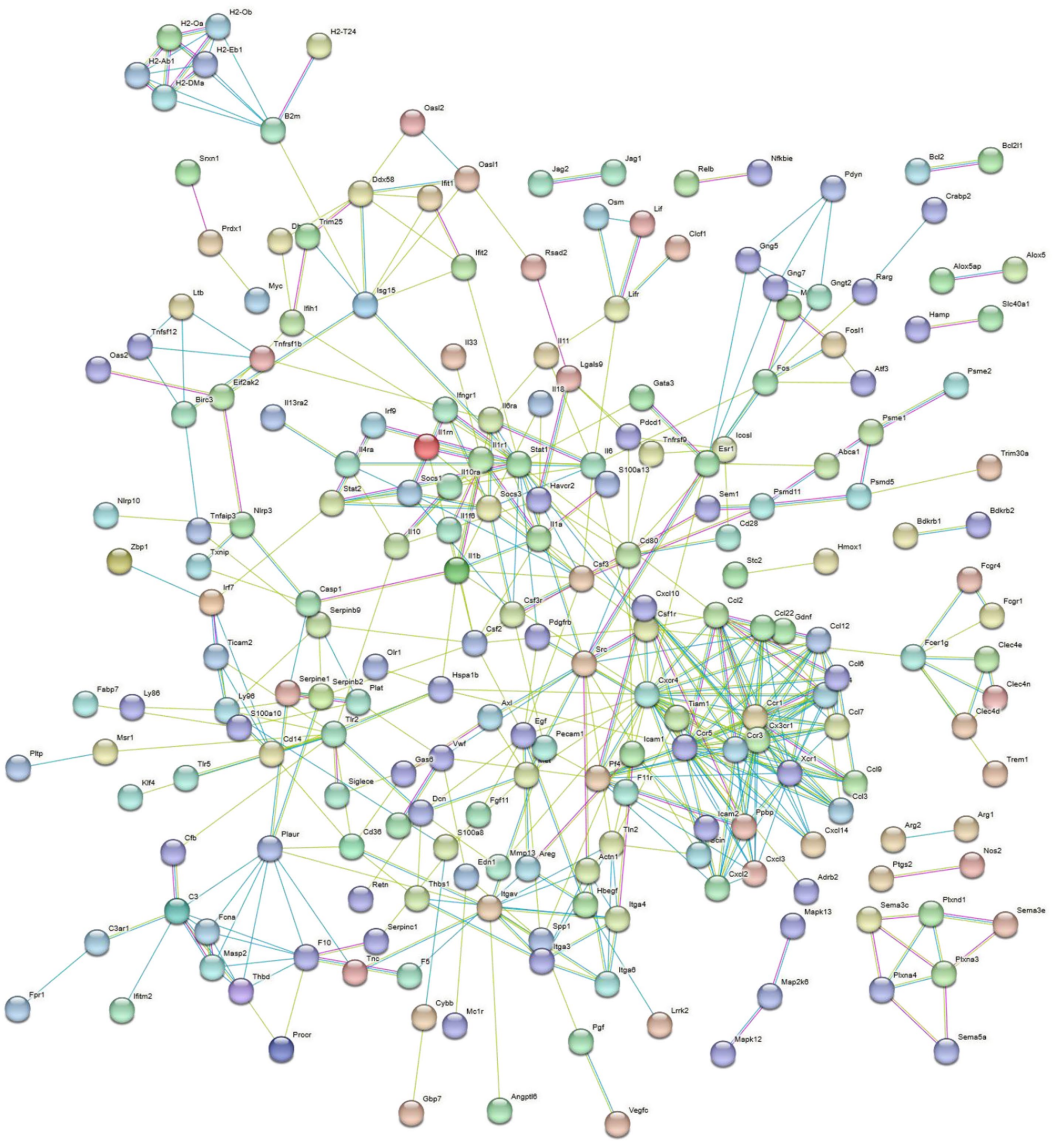
PPI analysis of immune related DEGs in the control group and the BVDV single infection group. The network was generated using the STRING database (https://string-db.org/).

One thousand nine hundred and twenty-six DEGs were found between the BVDV single-infection and the control groups. KEGG pathway enrichment analysis revealed that the differentially expressed genes were significantly enriched in immune-related signaling pathways, including the C-type lectin receptor signaling pathway, the NOD-like receptor signaling pathway, and the Cytokine-cytokine receptor interaction pathway (Figure 5). Therefore, BVDV infection likely triggered robust host defense responses in RAW264.7 cells, primarily through pathogen recognition via pattern recognition receptors (PRRs) and the subsequent activation of inflammatory signaling. Immune-related gene screening was conducted on 1,823 DEGs using the InnateDB, ImmPort, and KEGG Immune System databases, of which 308 DEGs were identified. PPI network analysis was performed on these differentially expressed genes. The genes *Casp1* (Caspase 1), *Cxcr4* (C-X-C chemokine receptor type 4), *Stat1* (Signal transducer and activator of transcription 1), and *Ccl2* (C-C motif chemokine ligand 2) may serve as key genes for the host immune response to BVDV infection (Figure 6).

### 3.6 BVDV co-infection reshapes the immune response induced by the *Brucella abortus* A19 vaccine strain in RAW264.7 cells

**Figure 7:**
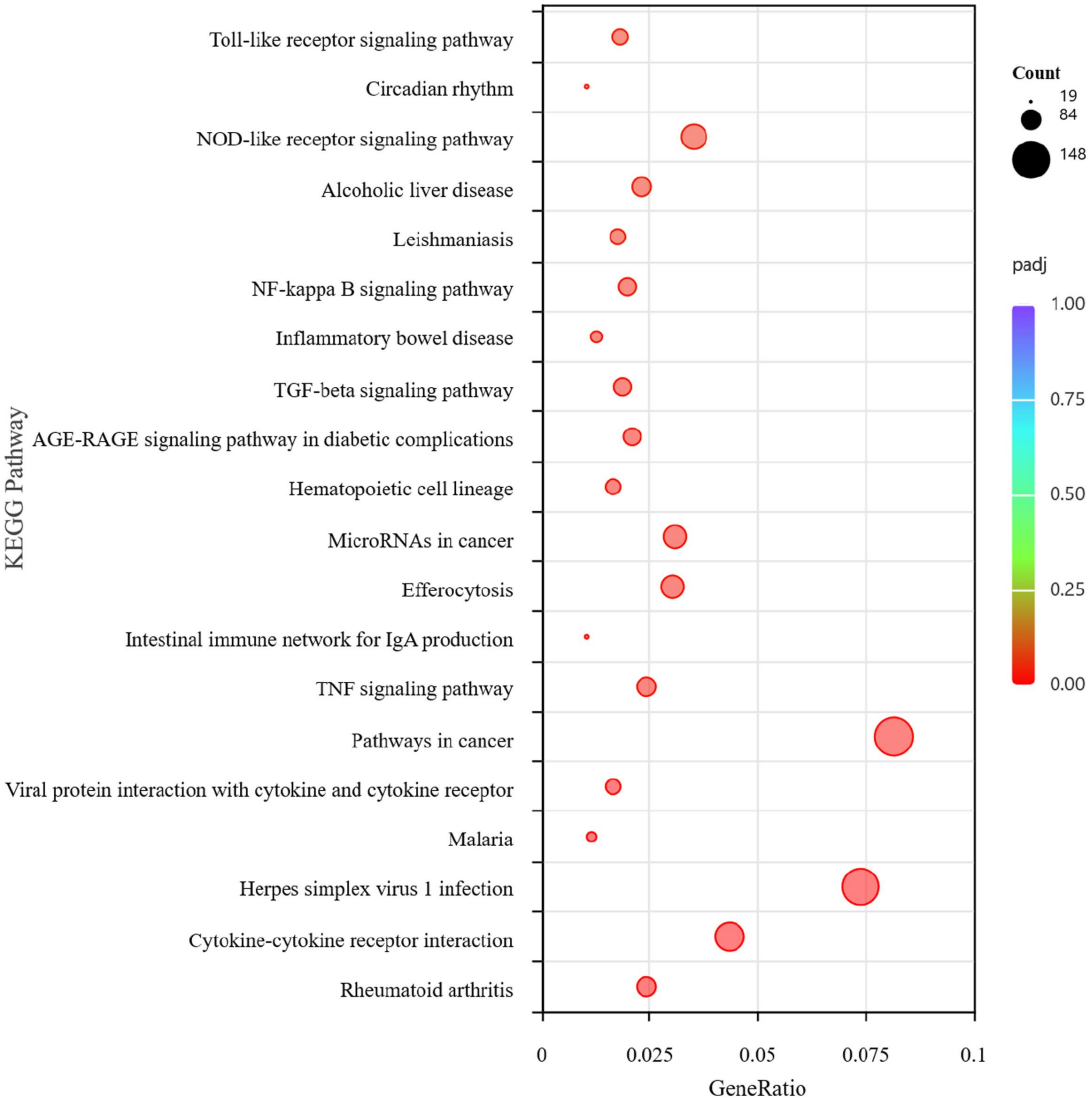
KEGG pathway analysis of DEGs in the BVDV+A19 co-infection group and the *B. abortus* A19 single infection group.

**Figure 8:**
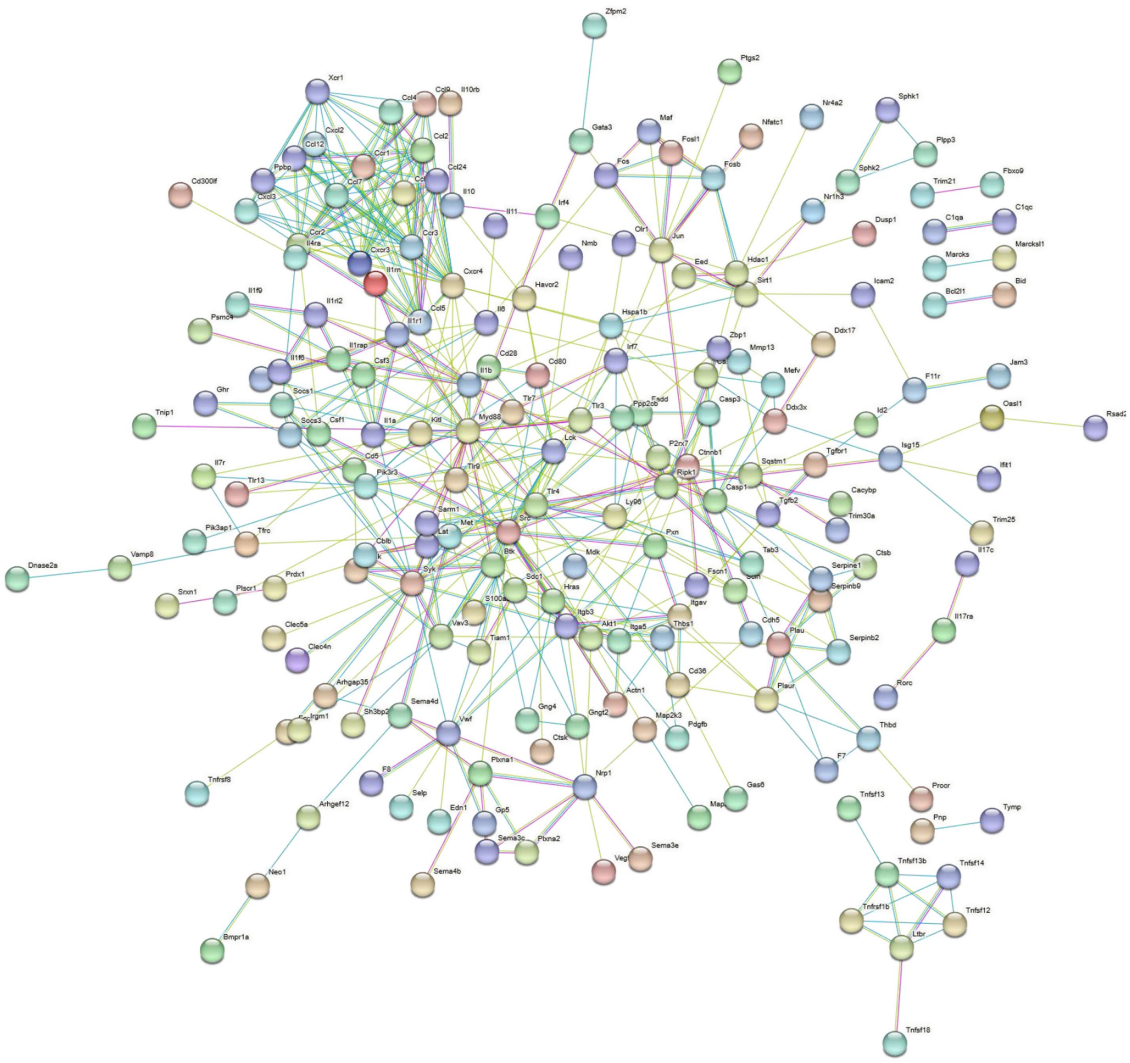
PPI analysis of immune related upregulated DEGs in the BVDV+A19 co-infection group and the *B. abortus* A19 single infection group. The network was generated using the STRING database (https://string-db.org/).

**Figure 9:**
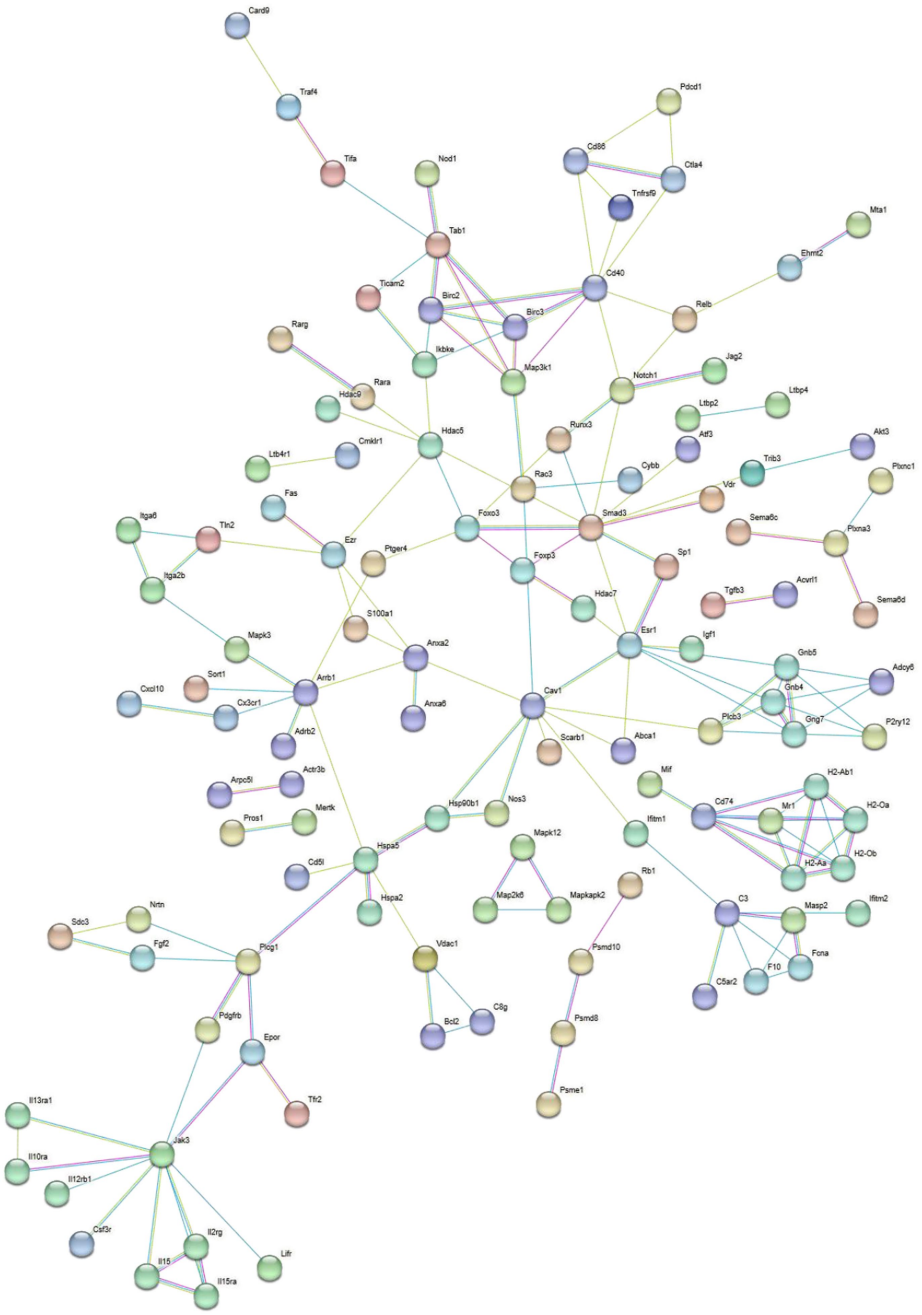
PPI analysis of immune related the downregulated DEGs in the BVDV+A19 co-infection group and the *B. abortus* A19 single infection group. The network was generated using the STRING database (https://string-db.org/).

Overall, 4,810 DEGs were found between the *B. abortus* A19 single-infection and BVDV+A19 co-infection groups. KEGG pathway analysis revealed that typical chronic inflammation and autoimmune disease pathways, such as rheumatoid arthritis, inflammatory bowel disease, and alcoholic liver disease, were also significantly enriched (Figure 7). These results indicate that BVDV infection altered the transcriptome of RAW264.7 cells induced by the *B. abortus* A19 vaccine strain. According to the InnateDB, ImmPort, and KEGG Immune System databases, 2,344 upregulated and 2,466 downregulated DEGs were screened for immune-related genes, and 284 upregulated and 217 downregulated immunity-associated DEGs were analyzed using PPI networks. The results showed that among these upregulated DEGs, *Ccl2* (myeloid differentiation primary response protein), *Ccr2* (C-C chemokine receptor type 2), and *Tlr4* (Toll-like receptor 4) may be key genes (Figure 8). Among the downregulated DEGs, *Cd74* (H-2 class II histocompatibility antigen gamma chain), *Smad3* (mothers against decapentaplegic homolog 3), and *Il15* (interleu-kin-15) may be key genes (Figure 9). These results indicate that BVDV co-infection affects the signaling pathways induced by the *B. abortus* A19 vaccine strain in RAW264.7 cells.

### 3.7 BVDV infection reverses *Brucella abortus* A19-induced immune gene expression

**Figure 10:**
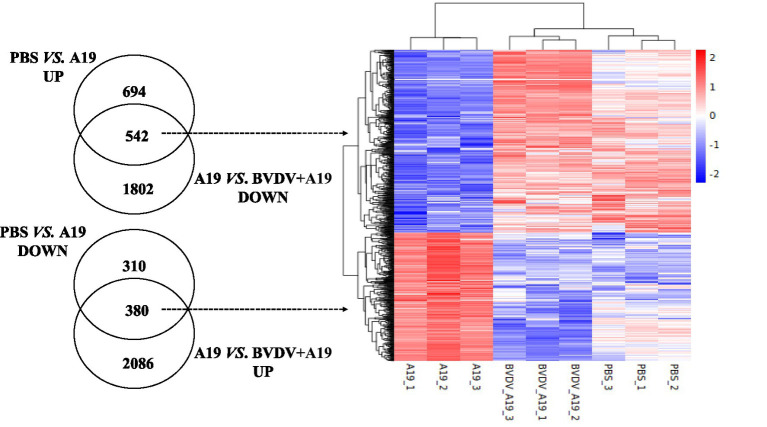
Overview of the DEGs after different treatments.

Venn diagram analysis revealed 1,047 overlapping DEGs. Among these, 922 showed an opposite expression pattern, with 542 being upregulated under *B. abortus* A19 stimulation and downregulated under BVDV+A19 co-stimulation. In addition, 380 DEGs showed an opposite expression trend (Figure 10).

**Figure 11:**
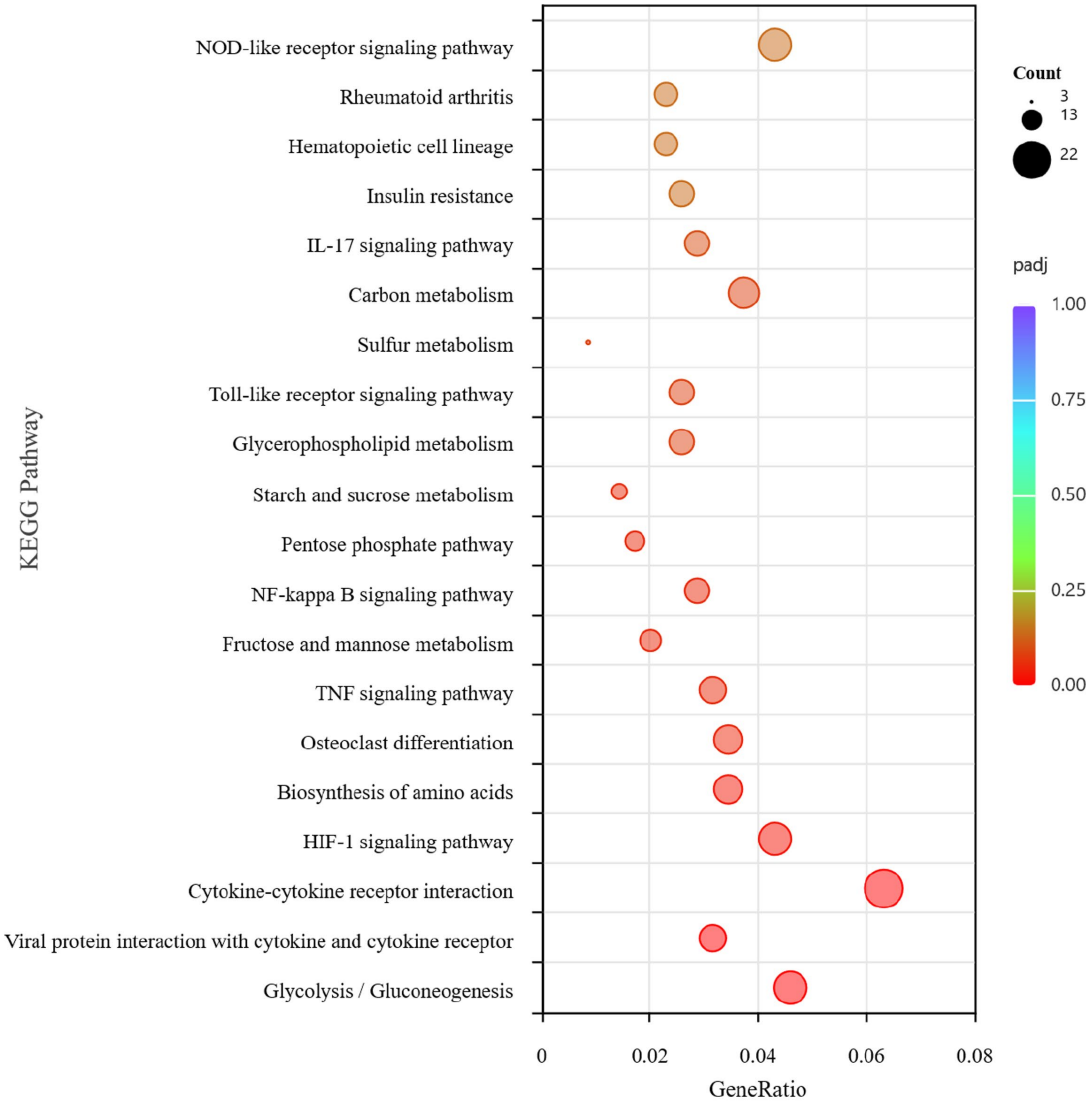
KEGG enrichment analysis of the DEGs overlapping between the two DEGs groups.

**Figure 12:**
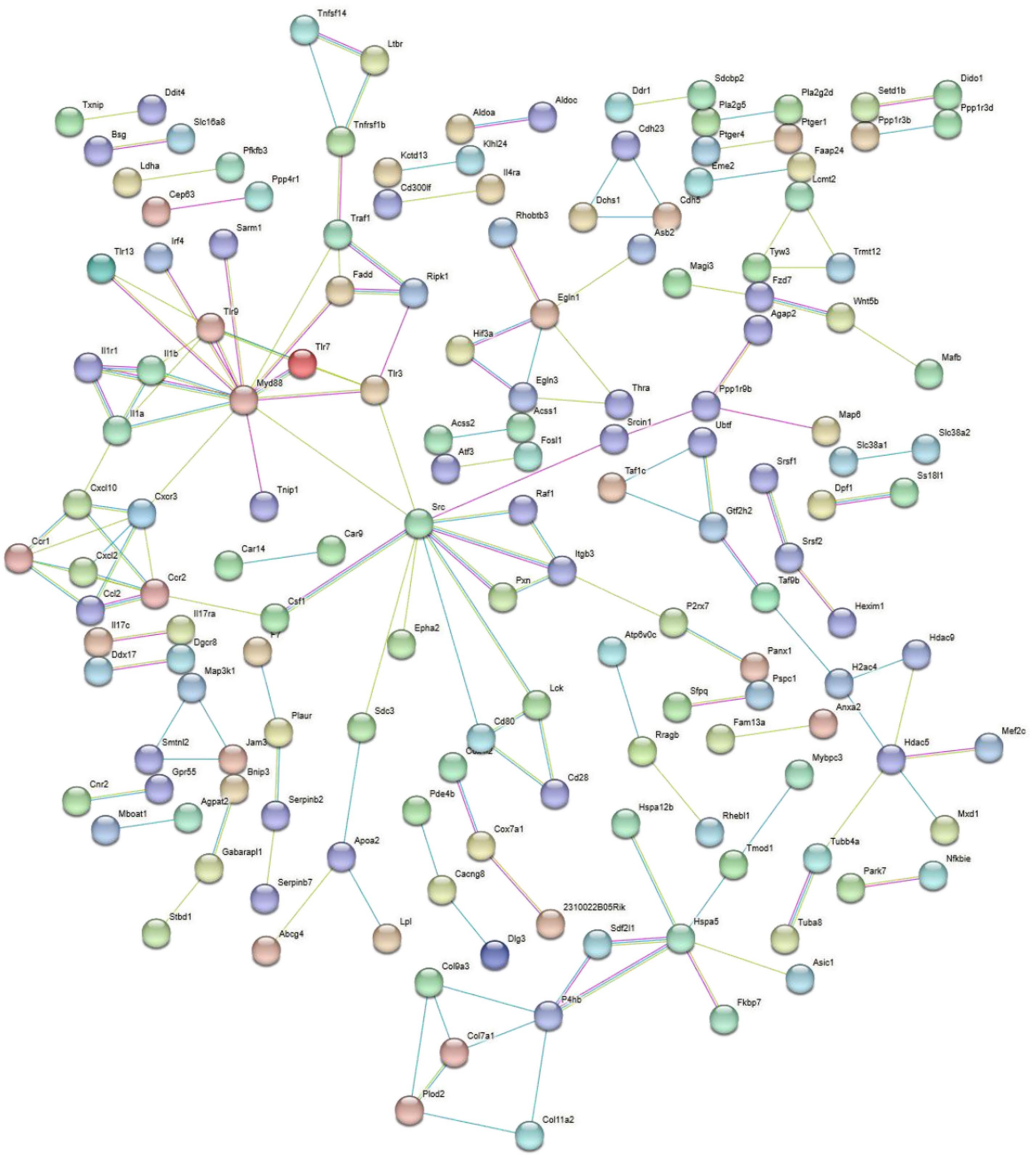
PPI analysis of the DEGs overlapping between the two DEGs groups.

KEGG analysis was performed on 922 DEGs with reverse expression, identifying rheumatoid arthritis as a significantly distinct signaling pathway, including *Cxcl2* (C-X-C motif chemokine ligand 2), *Il1a* (interleukin-1 alpha), *Il1b* (interleukin-1 beta), *Csf1* (processed macrophage colony-stimulating factor 1), *Atp6v0c* (ATPase H + Transporting V0 Subunit C), *Cd80* (Cluster of Differentiation 80), *Cd28* (Cluster of Differentiation 28), and *Ccl2* (Figure 11). Additionally, *Src* (neuronal proto-oncogene tyrosine-protein kinase) and *Myd88* are central network BVDV-altered genes in the PPI, which are involved in the immune response induced by the *B. abortus* A19 vaccine strain in RAW264.7 cells (Figure 12). Among the 922 genes exhibiting reverse expression compared to that of the control group, the immune-related genes *Ifit1* (interferon-induced protein with tetratricopeptide repeats 1), *Il1a*, and *Il17c* (interleukin-17C) were downregulated in the *B. abortus* A19 single-infection group. Compared with the *B. abortus* A19 single-infection group, BVDV infection significantly upregulated gene expression.

### 3.8 RNA-seq data is consistent with the qRT-PCR results

To validate the transcriptomic changes identified by RNA-seq, we performed quantitative real-time PCR (qRT-PCR) on a panel of 13 genes involved in immune response and transcriptional regulation, including *Irf-7*, *Irf-9*, *Fosl1*, *Fosb*, *Fos*, *Runx3*, *Nfyc*, *Nr1h3*, *Nfatc1*, *Gata3*, *Plscr1*, and *Jun*. The log₂ (fold change) values from both assays were compared to assess the concordance between the two methods.

**Figure 13:**
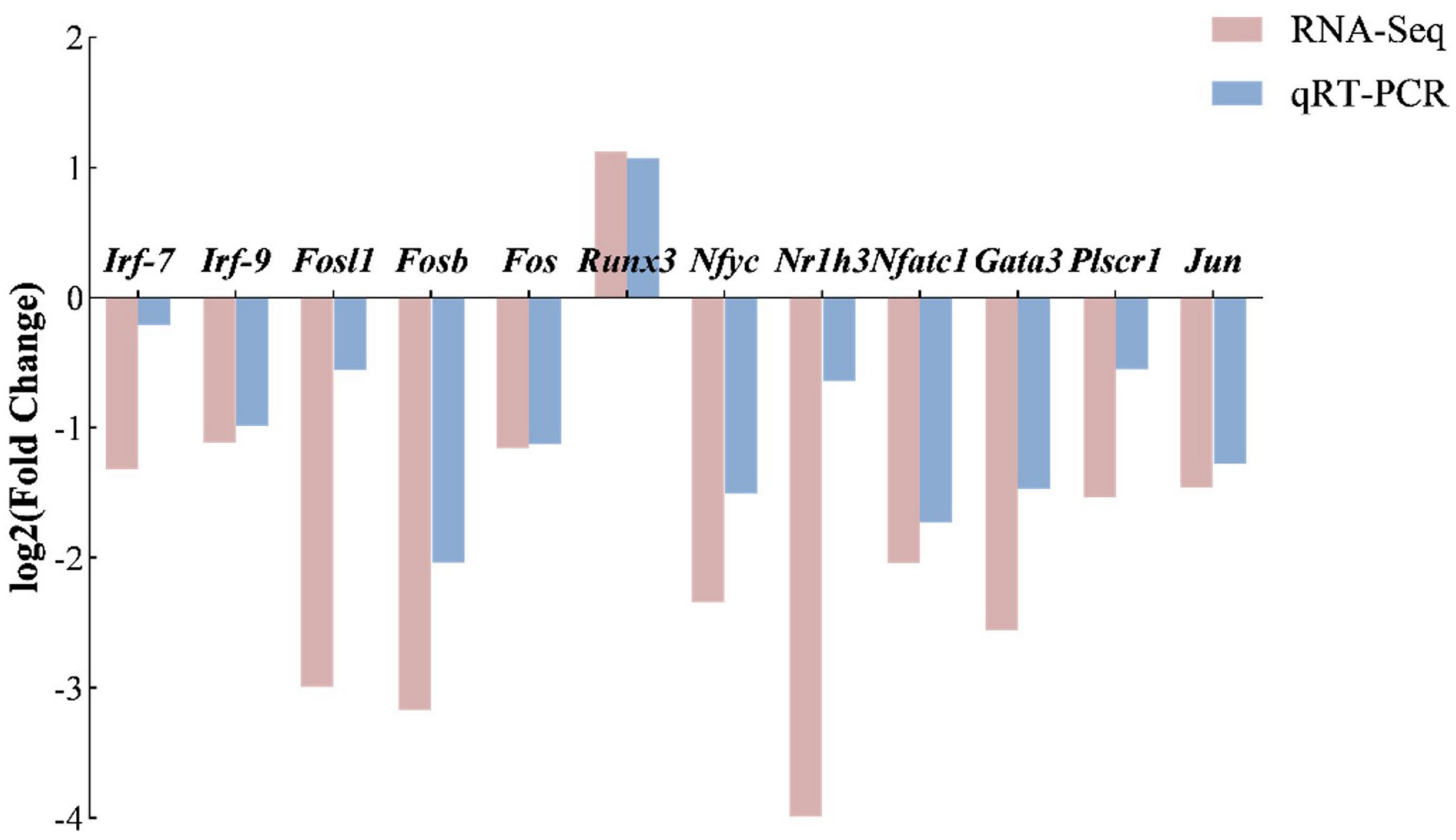
The verification of RNA-Seq results by qRT-PCR, between the *B. abortus* A19 and BVDV+A19 groups. The samples were analyzed in triplicate by qRT-PCR, and fold-changes in gene expression were calculated by 2^−ΔΔCT^ methods with B-actin as a reference gene.

As shown in the bar chart (Figure 13), the expression trends of the 12 selected genes were highly consistent between RNA-seq and qRT-PCR. Runx3 was upregulated in both platforms, while the remaining 11 genes were downregulated. Notably, the magnitude of fold change tended to be greater in RNA-seq than in qRT-PCR, which may reflect inherent differences in the sensitivity and dynamic range of the two technologies. Overall, these results validate the reliability of our RNA-seq data. Meanwhile, to better understand the biological roles of the selected genes, functional annotation of the qPCR-validated genes was summarized in Table 5.

**Table 5:** Functional annotation of qPCR-validated genes.

Gene name	Gene type	Core biological functions
Irf-7	Transcription factor (Interferon regulatory factor family)	Master regulator of type I interferon (IFN-α/β) signaling; critical for antiviral innate immunity and inflammation
Irf-9	Transcription factor (Interferon regulatory factor family)	Forms ISGF3 complex with STAT1/2 to mediate type I IFN signaling; drives antiviral gene expression and immune cell differentiation
Fosl1	Transcription factor (Fos family, AP-1 subunit)	Forms AP-1 heterodimers with Jun proteins; regulates cell proliferation, differentiation, macrophage polarization, and tumor microenvironment remodeling
Fosb	Transcription factor (Fos family, AP-1 subunit)	Core AP-1 subunit responding to calcium, growth factors, and stress
Fos	Transcription factor (Fos family, AP-1 subunit)	Immediate early gene; forms AP-1 complexes to regulate proliferation, apoptosis, inflammation, and osteoclast differentiation
Runx3	Transcription factor (Runt-related family)	Tumor suppressor via inhibiting oncogenes and TGF-β/Wnt pathways; regulates T-cell differentiation, DC maturation, and neurodevelopment
Nfyc	Transcription factor (NF-Y complex γ-subunit)	Core subunit of NF-Y heterotrimer; binds CCAAT boxes to regulate cell cycle, metabolism, and development; dysregulation linked to tumorigenesis
Nr1h3	Nuclear receptor (Liver X receptor α)	Key sensor of cholesterol and lipid metabolism; promotes reverse cholesterol transport and exerts anti-inflammatory effects in macrophages
Nfatc1	Transcription factor (Nuclear factor of activated T cells family)	Calcineurin-dependent TF; critical for T-cell activation, osteoclast differentiation, and cardiac valve development; modulates cytokine and apoptotic gene expression
Gata3	Transcription factor (GATA family)	Master regulator of Th2 cell differentiation and IL-4/5/13 expression; involved in mammary gland, kidney, and neuronal development; dysregulation linked to immune diseases and cancer
Plscr1	Phospholipid scramblase	Mediates membrane phospholipid rearrangement and phosphatidylserine externalization during apoptosis; acts as an ISG with broad antiviral activity
Jun	Transcription factor (Jun family, AP-1 subunit)	Forms AP-1 complexes with Fos proteins; regulates proliferation, apoptosis, inflammation, and tumor progression downstream of MAPK signaling

## 4 Discussion

BVDV infection causes substantial economic losses to the global cattle industry (27). It primarily causes clinical symptoms, such as immunosuppression, reproductive disorders, and mucosal damage in cattle. BVDV can lead to persistent infections and fatal mucosal diseases (28). Additionally, the virus can form a cyclic infection chain in cattle herds through both vertical and horizontal transmission (29). However, the specific molecular mechanisms and signaling pathways mediated by the immune response induced by the *B. abortus* A19 vaccine after BVDV infection in the host have not been fully elucidated.

In this study, we investigated the effect of *B. abortus* A19 infection on the immune response of RAW264.7 cells using transcriptome analysis. In total, 1,926 DEGs were identified by comparing the control and infected groups. KEGG enrichment analysis revealed that the significantly enriched signaling pathways were primarily involved in immune responses, including cytokine-cytokine receptor interaction and Toll-like receptor, NF-κB, and TNF signaling pathways, among others. Within these pathways, we observed: some chemokines, such as *Ccl3* (C-C motif chemokine 3), *Ccl4* (C-C motif chemokine 4), *Ccl5*, and *Cxcl10* (C-X-C motif chemokine 10), being upregulated, suggesting an active attempt to recruit immune cells to the infection site. However, most DEGs in the Toll-like receptor signaling pathway were downregulated, including *Tlr7*, *Tlr9* (Toll-like receptor 9), *Cd80*, *Cd86*, and *Myd88*. This pattern indicates that while *B. abortus* A19 triggers chemotactic signals, it simultaneously suppresses the downstream recognition and co-stimulatory machinery, potentially as an immune evasion strategy. In addition, *Myd88*, *Src*, and *Ccl2* were identified as key genes in the cellular response to *B. abortus* A19 using PPI network analysis. *Myd88* is the central adapter of the TLR/IL-1R signaling pathway and core of innate immunity (30). *Src* is a non-receptor tyrosine kinase that regulates cell adhesion, migration, proliferation, and survival (31). *Ccl2* recruits monocytes and macrophages to sites of inflammation and tissue damage (32). *Src* and *Ccl2* are crucial for immune cell migration and activation of immune cells (33, 34). Notably, all three genes were downregulated in our dataset, suggesting a coordinated suppression of the TLR-*Src*-*Ccl2* axis. This coordinated downregulation may impair both the initiation (*Myd88*), the execution (*Src*), and the amplification (*Ccl2*) of the host immune response, favoring bacterial persistence. According to previous reports, Brucella inhibits multiple immune-related signaling pathways, thereby establishing persistent infections, which is consistent with our findings (35, 36).

There were 4,810 DEGs between the BVDV+A19 co-infection and *B. abortus* A19 single-infection groups, including 2,344 upregulated and 2,466 downregulated DEGs. We screened a subset of immune-related genes from these DEGs. The InnateDB, ImmPort, and KEGG Immune System databases were used to define the immune genes. In comparison, we identified 284 upregulated and 217 downregulated immune-related DEGs for subsequent PPI network construction. PPI network analysis revealed that these immune-related genes formed four interconnected functional clusters, suggesting that BVDV co-infection orchestrates a multi-faceted modulation of the host immune response rather than targeting isolated pathways. The four clusters comprised: (1) inflammation and cytokine signaling, (2) chemokine-receptor interaction network and immune-related DEGs, (3) interferon-associated antiviral response and apoptosis, and (4) stress response. Notably, these clusters were not independent, cross-cluster interactions were evident, particularly between the apoptosis-related genes in cluster 3 and the inflammatory genes in cluster 1 (CASP3 and TNFRSF1B connecting both clusters) (37), indicating a coordinated regulation of inflammation and cell death—a hallmark of programmed cell death pathways such as pyroptosis and necroptosis. Within the cell death-associated gene set, we identified a functional network centered on the interplay between apoptosis, necroptosis, and inflammasome signaling. Key genes included FADD and RIPK1, which are central to the apoptosis-necroptosis switch (38). CASP3 and CASP4, mediating apoptotic and pyroptotic cell death, respectively (39, 40); and ZBP1, a sensor that can trigger both apoptosis and necroptosis in response to viral infection (41). The concurrent upregulation of these genes suggests that BVDV co-infection may activate multiple, interconnected cell death pathways simultaneously, potentially amplifying immune cell loss and tissue damage. Notably, TNFRSF1B (42), RIPK1 (43), and CASP8 (44) also participate in NF-κB activation, linking cell death signaling to inflammatory responses. This dual role raises the possibility that BVDV co-infection exploits the crosstalk between inflammation and cell death to create a microenvironment that favors viral persistence while evading complete immune clearance. Collectively, these findings indicate that BVDV co-infection does not simply upregulate individual cell death genes but rather reconfigures the regulatory network connecting inflammation, apoptosis, and necroptosis.

Downregulated immune-related DEGs were widely involved in immune regulation, cell signaling transduction, antigen presentation, inflammatory responses, and apoptosis regulation. The significant downregulation of MHC class II antigen presentation-related genes, such as *Cd74* (H-2 class II histocompatibility antigen gamma chain), *H2-Aa* (H-2 class II histocompatibility antigen), and *H2-Ab1* (H-2 class II histocompatibility antigen), suggests that BVDV co-infection may impair antigen presentation ability, thereby affecting T cell activation (45). Simultaneously, the expression of cytokine receptor genes was de-creased, such as *Il10ra* (interleukin-10 receptor subunit alpha), *Il12rb1* (interleukin-12 receptor subunit beta-1), and *Il15ra* (soluble interleukin-15 receptor subunit alpha), indicating that cytokine-mediated immune cell–cell communication is obstructed (46). In addition, the decrease in *Jak3* (tyrosine-protein kinase) affected the transmission of various cytokine signaling pathways (e.g., *IL-2* and *IL-15*), further weakening lymphocyte proliferation and activation (47). According to PPI interaction network analysis, downregulated immune-related DEGs, such as *Arrb1* (beta-arrestin-1), *Cav1* (caveolin-1), and *Smad3*, may be key genes affecting the immune protective effect of the *B. abortus* A19 vaccine. *Arrb1* interacts with multiple GPCR signaling pathways and MAPK pathway members, and its downregulation may affect G protein-coupled receptor-mediated immune cell chemotaxis and activation (48, 49). *Cav1* is a membrane microstructure protein that interacts with genes such as *Nos3* (nitric oxide synthase) and *Abca1* (phospholipid-transporting ATPase). It participates in the regulation of inflammation and cholesterol metabolism, and its de-creased expression may affect the stability and function of the cell membrane signaling platform (50, 51). *Smad3* is a key intracellular signal transduction protein and transcription regulatory factor, and is one of the most important effector molecules in the TGF-β signaling pathway. *Smad3* downregulation disrupts the critical signal transduction required for T cell differentiation and activation, leading to cellular immune dysfunction and decreased antigen presentation efficiency. In summary, BVDV co-infection may weaken the immune protective effect induced by the *B. abortus* A19 vaccine by downregulating the ex-pression of key immune-related genes, disrupting the structure and function of cell signaling networks, and inhibiting antigen presentation, cytokine signaling, and cell survival mechanisms. These findings provide a molecular basis for understanding the immune interference mechanism of BVDV in mixed infections, and a theoretical basis for subsequent vaccine optimization and the design of combined immunization strategies.

Pathological changes in splenic lymphocyte necrosis were observed in the spleen of an animal model co-infected with BVDV and *B. abortus* A19. To reveal the potential immunological mechanisms at the molecular level, we analyzed the changes in immune-related DEGs in the BVDV+A19 co-infection and *B. abortus* A19 single-infection groups. Analysis of upregulated immune-related DEGs revealed the significant activation of pro-inflammatory signaling pathways. Pro-inflammatory cytokine genes, such as *Il1b*, *Il6*, and *Il33*, were significantly upregulated, directly inducing inflammation and disrupting microenvironmental homeostasis (52–54). As a pivotal molecule in the inflammasome pathway, the upregulation of *Casp1* expression indicates the activation of pyroptosis (55). In addition, increased expression of chemokines (*Ccl3*, *Ccl4*, and *Ccl5*) and their receptors (*Cxcr3* and *Ccr1*) may lead to excessive infiltration of immune cells into the spleen, resulting in lymphocyte necrosis (56). Analysis of downregulated immune-related DEGs showed that the protective and anti-inflammatory mechanisms of cells were significantly weakened, and downregulation of the anti-apoptotic protein Bcl2 reduced the survival threshold of lymphocytes under stress (57). Downregulation of the key molecule *Il10ra* in negative immune regulation weakened the physiological constraints on pro-inflammatory responses, leading to excessive enhancement of inflammatory responses mediated by *Il1b* and *Il6* (58). Simultaneously, downregulation of the endoplasmic reticulum stress-protective molecule Hspa5 leads to the dysfunction of endoplasmic reticulum protein folding, exacerbating the unfolded protein response under infectious stress, thereby weakening cell survival ability (59). In summary, mixed infections form a highly pro-inflammatory and protective microenvironment in the spleen. Specifically, pro-inflammatory signals and cell pyroptosis pathways are strongly activated, however, key survival-promoting and homeostasis-maintaining molecular functions are impaired. The combined effect of enhanced “pro-necrosis signal” and weakened “survival mechanism” constitutes the core molecular pathological basis of splenic lymphocyte necrosis, providing a powerful molecular explanation for the clinical pathological observations.

BVDV co-infection altered the transcriptional response induced by *B. abortus* A19 single infection. Overall, 922 genes showed reverse expression in the two DEGs groups, and KEGG analysis showed that the reverse-expressed genes were significantly enriched in multiple pathways related to immune regulation, inflammatory responses, and metabolic reprogramming. Cytokine-cytokine receptor interaction, TNF signaling, NF-kappa B signaling, and key immune-related pathways, such as the Toll-like receptor signaling pathway, were significantly enriched, indicating that BVDV infection may interfere with innate and adaptive immune responses activated by the *B. abortus* A19 vaccine. In addition, metabolic pathways such as glycolysis/gluconeogenesis and amino acid biosynthesis were significantly enriched, indicating that BVDV infection may support its replication by reprogramming the metabolic state of host cells and affect the function and differentiation of immune cells. Through PPI network analysis, *Src* and *Hspa5* (member 5 of the heat shock protein family A) were identified as key genes regulating the response of RAW264.7 cells to the *B. abortus* A19 vaccine by BVDV. Studies have shown that a moderate inflammatory response helps the host clear intracellular pathogens; however, other studies have suggested that an excessive immune response may exacerbate tissue damage and pro-mote pathogen immune escape (60, 61). In this study, BVDV induced excessive inflammation and an immune response by upregulating the expression of genes, such as *Ifit1*, *Il1a*, and *Il17c*, thereby affecting the immune recognition and response ability of *B. abortus* A19 (62–64). In addition to the aforementioned genes, the reverse-expression genes included multiple genes related to metabolic reprogramming and immune regulation, such as *Ldha* (L-lactate dehydrogenase A chain), *Aldoa* (fructose-bisphosphate aldolase A), and *Pfkp* (ATP-dependent 6-phosphofructokinase). These genes were downregulated in the *B. abortus* A19 single-infection group and upregulated in the BVDV+A19 co-infection group, indicating that BVDV may affect the intracellular environment of Brucella by reshaping the host cell metabolic status and immune signaling network. The analysis results of the re-verse expression genes were consistent with the DEGs analysis conclusions of the *B. abortus* A19 single-infection and BVDV+A19 co-infection groups. This study reveals the regulatory effect of BVDV co-infection on the transcriptional profile of RAW264.7 cells induced by the *B. abortus* A19 vaccine, providing a new perspective for understanding the mechanism of viral-bacterial co-infection.

While this study provides novel insights into the transcriptional interference of BVDV on the cellular response to the *B. abortus* A19 vaccine in RAW264.7 macrophages, several limitations should be acknowledged. First, as a murine macrophage cell line, RAW264.7 cells may not fully recapitulate the physiological characteristics and immune responses of primary bovine macrophages, the natural host target of both pathogens. Second, our *in vitro* system lacks the complex multicellular interactions and tissue microenvironment present in living cattle. Therefore, while our findings reveal potential molecular mechanisms of immune interference, extrapolation of these results to direct conclusions about vaccine efficacy in the bovine host should be made with caution. Future studies utilizing bovine primary macrophages or *in vivo* challenge models in cattle are necessary to validate whether the observed transcriptional changes translate to altered vaccine protection against *Brucella abortus* under natural infection conditions.

## 5 Conclusion

This study demonstrates that in RAW264.7 murine macrophages, co-infection with BVDV alters the host cell transcriptional profile induced by the *B. abortus* A19 vaccine strain. These alterations include the disruption of cytokine signaling, inhibition of antigen presentation pathways, and an imbalance between pro-inflammatory responses and anti-apoptotic/homeostatic mechanisms. Our findings suggest that BVDV may interfere with the cellular immune response to the *B. abortus* A19 vaccine *in vitro*, revealing a potential mechanism by which viral co-infection could affect vaccine-induced immunity. However, further validation in bovine primary cells or *in vivo* models is required to confirm the relevance of these observations for vaccine efficacy in cattle.

## Data Availability

The datasets generated for this study have been deposited in the NCBI BioProject database under accession number PRJNA1435742.

## Footnotes

1
http://www.genome.jp

2
http://magicplusnovel.com

3
http://cn.string-db.org

4
http://www.innatedb.ca/

5
https://www.immport.org/
